# COMP Report: CPQR Technical Quality Control Guidelines for Data Management Systems

**DOI:** 10.1002/acm2.12385

**Published:** 2018-07-01

**Authors:** Natalie Pomerleau‐Dalcourt, Parminder Basran

**Affiliations:** ^1^ Département de Physique Médicale Centre d'oncologie Dr. Léon‐Richard Réseau de Santé Vitalité Moncton NB E1C 8X3 Canada; ^2^ Department of Medical Physics British Columbia Cancer Agency – Vancouver Island Centre Victoria BC V8R 6V5 Canada

**Keywords:** data management systems, electronic, medical records, quality control guidelines, radiation therapy

## Abstract

The Canadian Organization of Medical Physicists (COMP), in close partnership with the Canadian Partnership for Quality Radiotherapy (CPQR) has developed a series of Technical Quality Control (TQC) guidelines for radiation treatment equipment. These guidelines outline the performance objectives that equipment should meet in order to ensure an acceptable level of radiation treatment quality. The TQC guidelines have been rigorously reviewed and field tested in a variety of Canadian radiation treatment facilities. The development process enables rapid review and update to keep the guidelines current with changes in technology (the most updated version of this guideline can be found on the CPQR website). This particular TQC details recommended quality control testing of radiation data management systems.

## 1 INTRODUCTION

The Canadian Partnership for Quality Radiotherapy (CPQR) is an alliance among the three key national professional organizations involved in the delivery of radiation treatment in Canada: the Canadian Association of Radiation Oncology (CARO), the Canadian Organization of Medical Physicists (COMP), and the Canadian Association of Medical Radiation Technologists (CAMRT). Financial and strategic backing is provided by the federal government through the Canadian Partnership Against Cancer (CPAC), a national resource for advancing cancer prevention and treatment. The mandate of the CPQR is to support the universal availability of high quality and safe radiotherapy for all Canadians through system performance improvement and the development of consensus‐based guidelines and indicators to aid in radiation treatment program development and evaluation. The development of the individual TQC guidelines is spearheaded by expert reviewers and involves broad stakeholder input from the medical physics and radiation oncology community.^1^

This document contains detailed performance objectives and safety criteria for Data Management Systems. Please refer to the overarching document Technical Quality Control Guidelines for Canadian Radiation Treatment Centers^2^ for a programmatic overview of technical quality control, and a description of how the performance objectives and criteria listed in this document should be interpreted.

All information contained in this document is intended to be used at the discretion of each individual center to help guide quality and safety program improvement. There are no legal standards supporting this document; specific federal or provincial regulations and license conditions take precedence over the content of this document.

## 2 SYSTEM DESCRIPTION

The fundamental definition of a data management system (DMS) has not changed since the publication of the Canadian Association of Provincial Cancer Agencies (CAPCA) quality control document for data management systems in 2008: a DMS is the information infrastructure which is directly related to the planning, delivery, quality assurance, and archival of patient treatments. In its simplest incarnation, a DMS can be a single computer. However, in the typical radiation treatment clinic, a DMS is comprised of many separate entities or systems that manage, store, and exchange information of many types and formats via various methods and protocols. The level of complexity of computer systems in radiation oncology clinics has seen a tremendous increase over the past several years. In part, this increase is due to the evolution of radiation treatment technology — the increasing complexity of treatment delivery systems which themselves contain multiple computerized systems, the ongoing evolution of onboard imaging systems, the increased variety and quantity of diagnostic and simulation imaging studies involved in the planning process, and the ever‐increasing evolution and scope of record/verify electronic medical record systems. The ongoing transition of many clinics toward a paperless or “paper‐light” environment is even further increasing the quantity and variety of data stored and managed by computerized systems in the clinic. And of course, beyond the walls of our clinics, the world of information technology and data management is expanding at a relentless pace — so that the hospital infrastructure and systems that often form the backbone of our radiation clinics’ data management systems are also evolving rapidly.

A comprehensive quality assurance program for a DMS should consider all of the separate components in the DMS, the exchange of data between components, and the procedures governing that exchange. Accordingly, the program could have three general categories:

1. Quality assurance of computerized systems: performance and functionality of each individual component in the DMS, data integrity within each component;
2. Quality assurance of data exchange: data exchange between components in the DMS (multiple formats, multiple protocols, via interface, or manual data transfer); and
3. Quality assurance of procedures (including data entry and data interpretation).

Key features of a quality assurance program should include: assembling a multidisciplinary team with regular meetings and clearly established roles and responsibilities; project management of scheduled upgrades and systematic tracking and evaluation of hardware and software failures and issues, and subsequent root‐cause analysis.

Each radiation treatment clinic's DMS is unique, making it impossible to prescribe a universal or one size fits all quality assurance program. Instead, this guidance document offers a step‐by‐step approach to aid the medical physicist in designing a tailored, context‐specific quality assurance program for each unique DMS (see Appendix 1). The lists of test categories included in Tables 1 and 2 and the specific tests detailed in Section 5 are meant to be comprehensive but not prescriptive, serving as a recipe box from which the qualified medical physicist can select the appropriate tests for their unique DMS. Furthermore, testing frequencies must be established based on in‐depth knowledge of the relevant clinical processes — the suggestions made in this document serve as a reasonable baseline that should be modified to suit a given DMS. Some of the tests chosen for the DMS quality assurance program will likely be the responsibility of IT personnel. Others will be the responsibility of the medical physicist. It is probable that some of the tests will require collaboration and input from the appropriate vendor. The approach described here is adapted in part from that suggested by Report 93 of the Institute of Physics and Engineering in Medicine (IPEM),^3^ incorporating suggestions from the preliminary recommendations of the American Association of Physicists in Medicine's (AAPM) TG 201^4^, ^5^ as well as the existing CAPCA document for Data Management Systems. While not entirely in scope, this document may provide some guidance for those who are in the process of developing a new center's DMS or merging an existing DMS within an existing hospital's IT infrastructure.

**Table 1:** Categories of quality control tests for DMS data links, components and procedures

Designator	Test	Performance
DMS data links		
L1	Data transfer integrity	Complete
L2	Data transfer integrity of images and imaging data	Complete
L3	Data transfer integrity of electronic documents	Complete
L4	Tests for failure	Complete
L5	Soak or endurance testing	Complete
DMS components		
C1	Performance tests	Complete
C2	Network tests	Compare to baseline
C3	Security tests	Complete
C4	Data integrity	Complete
C5	Tests for failure	Complete
C6	Machine readout checks	Complete
C7	Data capture	Complete
C8	General administrative checks	Complete
Procedures		
P1	End‐to‐end testing	Complete
P2	Review of clinical process maps	Complete
P3	Contingency plan review	Complete

**Table 2:** Overall quality control program tests for a general DMS similar to Figs. A1, A2, A3, A4, A5, A6

Designator	Test	Performance
Annual overall program		
O1	Quality assurance program review	Complete
O2	Review of hardware and software inventories and DMS system maps	Complete
O3	Review of audits (network traffic, user access, etc.)	Complete
O4	Scheduled upgrades	Complete

In many clinics, IT personnel will be responsible for physical networks and hardware, as well as existing software and hospital information management systems. Consequently, it is entirely possible that the medical physicist responsible for a radiation treatment clinic's DMS will not have the necessary resources, knowledge and/or access to design and implement the DMS quality assurance program on their own. In a comprehensive report,^3^ the IPEM highly recommends that management of the radiation treatment DMS be maintained by medical physicists, with the support of dedicated IT specialists. Another model requires responsible medical physicists with solid IT skills to act as application owners for all “medical device tier” systems — those clinical systems directly affecting patient care. The maintenance of physical and virtual networks and computers and associated software falls under the responsibility of IT personnel. For safe and effective delivery of radiotherapy, a high degree of collaboration and a certain degree of knowledge‐overlap is needed between the responsible medical physicist and IT personnel. This requires the assigned IT support staff to be on site.^4^ For further guidance regarding appropriate training for radiotherapy IT professionals, see Siochi et al., 2009, Information Technology Resource Management in Radiation Oncology.^4^

It is recognized that existing organizational structures vary widely between individual radiotherapy clinics. While in some cases, the IT specialists are members of the radiotherapy department, this is certainly not always the case. It is recommended that, as a minimum, consultation and ongoing collaboration with responsible IT specialists will be required. A multidisciplinary team should be established to share responsibility for the DMS quality assurance program. The roles and responsibilities of all team members must be clearly defined to ensure accountability.^5^ This collaboration will ensure that responsible IT personnel are aware of the details, complexities, and critical nature of the systems used for radiotherapy as well as ensure appropriate resources for management, testing, maintenance, security, trouble‐shooting, training, and support.^3^

## 3 TESTING TOOLS

### 3.A Software

While many of the tests suggested here can be executed manually using test data, certain categories of tests are better suited to automated testing, specialized testing tools exist that have the capability to manage, script, and automate soak or endurance tests, network performance tests, and/or data integrity checks. Examples include, but are not limited to, Microsoft Visual Studio Testing Tools and Services, AgileLoad, HeavyLoad, Solarwinds, and Inflectra SpiraTest. Some tools are designed for specific types of testing, whereas other more comprehensive software suites offer tools to script and execute tests based on a schedule and include project management components to store and track the results of each test.

In some cases, manual end‐to‐end testing with a large number of test scenarios can be resource‐intensive. Automated testing tools are available that mimic a user's interaction with a workstation by sending data as if a user were interacting with the environment. A set of test data can be constructed and should be chosen to include the range of clinically relevant scenarios. Network communication from a sending computer to a receiving computer can be replicated. Some testing software will record a user‐interaction and store it as a test script, which can then be modified and/or replicated. Certain types of tests cannot be fully automated and require user interaction. For these manual tests, the software can prescribe the specific steps a tester is required to follow, including test data and order, state the expected behavior at each step and allow the tester to document the results.

The integration of such testing tools in a clinic's DMS requires collaboration with IT personnel and the appropriate vendors. Also note that the timing of automated tests should include sufficient delays between executions to avoid placing an artificial load on the system (which could artificially produce errors).

### 3.B Checksums and data validation

A checksum is a type of redundancy check that can be used to evaluate data integrity following transmission across a network (or data link), or following any other manipulation that could introduce error. An algorithm is used to calculate binary or other values that represent the data packet that can be compared at the beginning and end of each test point. If the outputted values are not identical, then the data being tested have changed in some way. Checksums algorithms can be executed against various data, including very large data items, which are otherwise difficult or time‐consuming to compare. Usually using a cryptographic hash (or similar) function, given a specific data value, it will always produce exactly the same result. Further, there are other approaches that operate similarly to checksums, for example, Cyclic Redundancy Codes (CRC). Each has its benefits and weaknesses and the choice of tools must be evaluated based on reliability, cost, and criticality of the data/component/link being tested. These data validation methods are not usually necessary on a routine basis, but are useful when verifying the integrity of user‐specific data, such as treatment planning beam models during upgrades, software revisions, and maintenance releases.

### 3.C Virtualizations

Generally, virtualization refers to the creation of a virtual version of a device, such as a server, network storage device, operating system, application, or service. Virtualization should only be performed with the support of the appropriate vendors as not all DMS components lend themselves to virtualization. In some cases, DMS performance can be negatively impacted.

When testing virtualized environments such as, but not limited to Citrix virtualization, the unique architecture of these services needs to be taken into account. These environments reduce the need to manage many applications and/or desktop environments across multiple workstations and operating systems and thus allow for workstations that span multiple network security domains (or other complex configuration differences) to access the applications or possibly entire virtual desktops through a single managed network port and protocol. Application virtualization relies on a locally installed receiver application that communicates from the remote workstation to the application server, where the application is running. Modern receiver applications generally are add‐ons to the workstation's web browser.

Virtualized environments require special testing considerations. The interface presented to users is virtualized — the user is presented with an image of the user interface of an application or desktop that is running on a remote server. This can introduce latency and lead to possible data input errors from the user, particularly in graphically intensive tasks such as contouring, registration, and dose display and manipulation. Virtual environments add complexity to automated tests that are meant to replicate user input — as most of these tests are configured to input data and submit as if from a user's workstation. Tests can be operated from within a virtual desktop through the virtualization services, but this does not replicate data passing through the “screen scraper” running on a local workstation.

Since remote applications are running from a server (virtualized or not), soak or endurance testing (see Designator L5 in Table 1) against the virtualization service is important — as if this service falters or fails, then any application in the DMS provided through the virtualization service may be impeded or unavailable.

When testing for failure (see Designator L4 in Table 1), the effect of a session timeout on an application when a user attempts to reconnect should be investigated. What is the state of the session and its data after the connection times out and is it recoverable? For the majority of applications on a modern virtualization environment, sessions should be recoverable for reconnection, within some period of time, and ideally harmonized with security and automatic disconnection settings (e.g., screen saver settings, automatic log‐off after inactivity).

In virtualized environments, imaging data are often compressed for faster data transfer. This compression and decompression can lead to reduced image quality. It is recommended that particular attention be paid to tests of data transfer integrity of images and imaging data (Table 1, Test designator L2).

Modern DMS infrastructures now virtualize servers as they offer lower cost of ownership, reduced footprints (hence reduced power and cooling requirements), more efficient data migration and backup/restores, and easier customization of hardware. They do, however, require additional layers of maintenance (hypervisors) and management which can affect overall DMS performance. Because they are often hosted through shared storage (SAN) environments not necessarily within the confines of the radiation oncology treatment center or location, they are susceptible to data loss due to a catastrophic events, large‐scale system degradation and outages. While the management of virtualized servers falls within the scope of IT professionals, given the risks to the integrity of the RO‐IT infrastructure, changes to virtualized server settings should be communicated to the DMS multidisciplinary team.

## 4 RELATED TECHNICAL QUALITY CONTROL GUIDELINES

In order to comprehensively assess data management system performance, additional guideline tests for integrated systems, as outlined in related CPQR Technical Quality Control (TQCs) guidelines must also be completed and documented, as applicable. Related TQC guidelines,^6^ available at cpqr.ca, include:

- CT Simulators
- Treatment Planning Systems
- Medical Linear Accelerators and Multileaf Collimators.

## 5 TEST TABLES

### 5.A Notes on tests for DMS links

Tests in this section are applicable to each data link (joining two computerized systems within the DMS). In addition to the tests suggested here, vendor recommendations for commissioning, acceptance testing, and regular quality assurance should be followed.

L1 **Data transfer integrity of general/demographics and treatment parameter data**
**Test:** For an appropriate range of clinical data, compare data sent vs. data received. Manual or automated tests can be performed using checksums or custom scripts using a bank of test data.
**Tolerances:** Different systems may have different levels of accuracy and also may have differing naming conventions. This can lead to errors — for example, due to rounding or truncation of data. Tolerances need to be established (whether zero or non zero) wherever data transfer occurs. To facilitate data transfer integrity tests, it is very helpful to construct data transfer tables for each data link. A data transfer table should include a full list of all parameters transferred, and the tolerances associated with the transfer. It is important to also be aware of any differences between the internal format or convention of the raw data and that displayed to the user. Data dictionaries can be valuable resources in the construction of these tables. Note that the selection of an appropriate range of clinical data is a nontrivial task requiring careful consideration of all clinically relevant treatment scenarios. A library of test cases can be constructed in the treatment planning system for use as needed and should be updated to reflect new and emerging treatment scenarios.
**Suggested frequency:** At commissioning, and following any change to the DMS components connected by the data link that could affect clinical data (including data formats, storage, display, tolerances, transfer protocols, etc.). A data transfer integrity test may be appropriate as part of routine patient quality assurance for certain clinical protocols — though it is likely this test will be of more limited scope. An example could be to compare critical treatment data given by the treatment console against a screen capture of approved plan data.

L2 **Data transfer integrity of images and imaging data**
  - **Geometric integrity (scale, accuracy)**
**Test:** Use a geometric test phantom with known dimensions and compare data before and after transfer, including appropriate scaling and/or processing.
**Suggested frequency:** At commissioning, and following any change to the DMS components connected by the data link that could affect imaging data (e.g., upgrade of cone beam CT [CBCT] software). This test is often part of existing quality assurance of imaging systems.
  - **Coordinate frame and patient orientation**
**Test:** Use a test phantom whose orientation is clearly identifiable. For all relevant orientations and positions, confirm that images are correctly transferred and interpreted.
**Suggested frequency:** At commissioning, and following any change to the DMS components connected by the data link that could affect imaging data (e.g., upgrade of CBCT software). This test is often part of existing quality assurance of imaging systems.
  - **Data transfer integrity of images and imaging data**
**Test:** Image quality: Using an appropriate phantom, evaluate image contrast, noise, and image intensity (e.g., HU value). Identify data degradation or distortion (e.g., due to compression). Compare values before and after image transfer. Compare against baseline or tolerance values as appropriate.
**Test:** File integrity: Using checksums or other tools, evaluate the integrity of the imaging files before and after transfer. Note that this test is required in addition to the above tests as it is possible for errors in integrity to be introduced that will not be visually apparent or detectable within the software used for image analysis.
**Suggested frequency:** At commissioning, and following any change to the DMS components connected by the data link that could affect imaging data (e.g., upgrade of CBCT software). This test is often part of existing quality assurance of imaging systems.

L3 **Data transfer integrity of electronic documents**
**Test:** Verify that transfer of electronic documents occurs as expected and that data format and integrity is maintained. Test should include all relevant document formats. Checksum or appropriate tools should be used in addition to visual inspection as errors can be introduced that will not inhibit document processing software from opening and manipulating the file.
**Suggested frequency:** At commissioning, and following any change to the DMS components connected by the data link that could affect the transfer of electronic documents [e.g., upgrade of electronic medical record (EMR) software].

L4 **Tests for failure**
  - **Out of limits data handling**
**Test:** Check that data exceeding limits of the receiving system are appropriately handled. This could include extra decimal points, null values, clinically irrelevant gantry or collimator examples, etc.
  - **Data corruption**
**Test:** Where possible, simulate data corruption and evaluate how this is handled by the receiving system. Include the handling of missing or truncated data, as may occur when data transfer is interrupted. Vendor input may be required to identify other relevant scenarios.
  - **System recovery from error state**
**Test:** Where possible, simulate error states and check that systems recover appropriately (and check for proper handling of data/data recovery). If mitigating steps are required to recover data, include these in your contingency plans. For example, some linear accelerators require the user to manually recover the record of delivered monitor units (MU) when a treatment beam is interrupted by a power failure or other unexpected system shut‐down.
    Where possible, tests A, B, and C should be automated to allow for large sets of sample data to be evaluated, recording the results of each execution and comparing against expected behavior.
**Suggested frequency** L4 A–C: At commissioning, and following any change to the DMS components connected by the data link that could affect clinical data (potentially use an appropriate subset of tests depending on the change to the system).

L5 **Soak or endurance testing**
**Test:** Test the ability of the system to handle large volumes of clinical data within a short time period. This test should be automated using testing tools as described later in this document. Soak or endurance testing should be performed after a full system backup and should not be performed during clinical operation. A set of interactions and inputs that reflect standard user input across the data link can be scripted. These are replicated in increasing test sizes so that the load on the system is stepped up over a measured time period. Performance counters should be collected during this time until the environment becomes unresponsive or until foreseeable data loads have been exceeded. Soak or endurance testing can be combined with data integrity tests as outlined in L1–L3 as data integrity results may be impacted by a higher system load, even if the environment appears to be operating properly (and is not yet unresponsive).
**Suggested frequency:** At commissioning or as needed (for example during troubleshooting for performance issues).

### 5.B Notes on tests for DMS components

Tests in this section apply to individual computerized systems within the DMS. In addition to the tests suggested here, vendor recommendations for commissioning, acceptance testing, and regular quality assurance should be followed. Some tests are also applicable to DMS data links — they are listed again here for completeness as they should be considered when implementing a new DMS component (or following an upgrade or other significant change).

C1 **Performance tests**
  - **Test:** Check accuracy of data transfer (using checksums, automated data transfer, redundancy checks, etc.) (see L1–L3 for details and suggested frequency).
  - **Test:** Monitor delay times (changes from baseline)
**Suggested frequency:** At commissioning and on an ongoing basis. Baseline values and thresholds should be established with the collaboration of the responsible IT personnel with input from vendors as appropriate.
  - **Test:** Monitor available memory, CPU usage (set thresholds)
**Suggested frequency:** At commissioning and on an ongoing basis. Lack of available memory can have unexpected impacts on performance and could lead to errors in data integrity. Automated tools exist to monitor system resources and alert system administrators when an established threshold is reached. If automated tools are not available, close monitoring is required.

C2 **Network tests**
  - **Test:** Validate accuracy and completeness of clinical data
**Suggested frequency:** At commissioning and quarterly.
  - **Test:** Monitor routing tables or routed and gated daemons.
  - **Test:** Connectivity tests [Digital Imaging and Communications in Medicine (DICOM), Echo, ping checks] between DMS components
  - **Test:** Monitor functionality of required services (depending on operating system)
  - **Test:** Monitor network speed/resource allocation (time required to transfer large test data between servers or computers, lag in console display)
**Suggested frequency:** C2 B–E: At commissioning, on an ongoing basis and during troubleshooting (of connectivity issues, for example). Baseline and threshold values depend strongly on the network design and infrastructure and should be established in collaboration with qualified IT personnel. Automated tools exist to monitor many aspects of network performance against established thresholds.

C3 **Security tests**
  - **Test:** Check for manufacturer security fixes (unless automatically provided by vendor)
  - **Test:** Maintain up‐to‐date list of applications, versions, patches, service packs, operating systems, etc.
  - **Test:** Maintain up‐to‐date antivirus software
  - **Test:** Adherence to pushed antivirus and other policy settings for standard and nonstandard computers
  - **Test:** Appropriateness of virus scan settings on specific workstations and servers (real‐time vs. scheduled for critical workstations and servers)
  - **Test:** Monitor user and system logs
  - **Test:** Evaluate and monitor physical and network boundaries including firewall settings
  - **Test:** Control user access permissions
  - **Test:** Physical hardware checks
**Suggested frequency:** C3 A–I: At commissioning and on an ongoing basis or as needed. Many of these tests may fall within the responsibility of IT personnel or will require input from IT personnel and/or vendors. Radiotherapy system components are highly sensitive to changes to security settings (virus scan settings, for example). The resulting loss of performance can be very difficult to troubleshoot without input and ongoing communication with IT personnel.

C4 **Data integrity**
  - **Test:** Validate accuracy and completeness of clinical data
**Suggested frequency:** Weekly or as appropriate based on criticality of data
  - **Test:** Data transfer tests (see Table 1 DMS Data Links tests)
  - **Test:** Automated data transfer checks (checksums, etc.)
  - **Test:** Validate backup/restore functionality (if applicable)
**Suggested frequency:** C4 A–D: At commissioning and following any change to the DMS component that could affect clinical data.

C5 **Tests for failure**
  - **Test:** Soak/endurance tests (see L5)
**Suggested frequency:** At commissioning
  - **Test:** Evaluate performance of critical systems following failure or simulated error states (system recovery, record locking, etc.).
**Suggested frequency:** At commissioning and following any change to the DMS component that could affect clinical data or on a regular basis where appropriate.

C6 **Machine readout checks**
**Test:** Compare display/readout to database. Note that the data visible to the user may be in a different format or following a different convention from that stored internally. Such conversions must be evaluated to ensure correct representation of data and appropriate degree of accuracy
**Suggested frequency:** At commissioning and following any change to the DMS component that could affect clinical data

C7 **Data capture**
**Test:** Validate capture of clinical data following user input. For example, couch settings as captured by treatment unit console and/or control software could be compared to treatment table readouts
**Suggested frequency:** At commissioning and following any change to the DMS component that could affect clinical data capture

C8 **General administrative checks**
**Test:** Check format/accuracy of printouts or other. Data included in printouts may follow a different format or convention and must be compared to ensure accuracy and correct representation
**Suggested frequency:** At commissioning and following any change to the DMS component that could affect clinical data

### 5.C Categories of tests for DMS procedures

P1 **End‐to‐end testing**
**Test:** Using carefully constructed, clinically relevant test cases, validate the complete clinical data chain from simulation to dose delivery. Test cases must be chosen to cover the full range of possible clinical situations.
**Suggested frequency:** At commissioning or following a change to any component of the DMS that is part of the routine clinical data flow. This type of testing is also valuable as part of the validation of a new treatment technique or, for some clinical protocols, as part of patient quality assurance. Regular end‐to‐end testing may be appropriate, especially in large systems with shared responsibility and management where changes to the DMS may occur without the responsible physicist's knowledge.
  Note that end‐to‐end testing alone is not sufficient — though the test result may show an error, it will not necessarily identify the source or cause of the error. In addition, end‐to‐end testing relies on test case construction. Without full testing of data transfer integrity between components in the DMS as outlined above, it is entirely possible to miss errors that will later impact clinical data.

P2 **Review of clinical process maps**
**Test:** Review existing documentation of clinical processes and update to reflect changes to DMS system components, links and/or procedures. Ideally, this test should be executed by a multidisciplinary team responsible for the DMS quality assurance program.
**Suggested frequency:** Annually or following a change to a DMS component that is part of the routine clinical data flow.

P3 **Contingency plan review**
  - **Test:** Review contingency procedures
**Suggested frequency:** Annually or following a change to backup and recovery systems within the DMS. Ideally, this test should be executed by a multidisciplinary team responsible for the DMS quality assurance program.
  - **Test:** Test alternative clinical data pathways.
**Suggested frequency:** Annually or following a change to backup and recovery systems within the DMS (during planned outages where possible).

## CONFLICT OF INTEREST

The authors have no conflicts of interest.

## Figures and Tables

**Figure A1:**
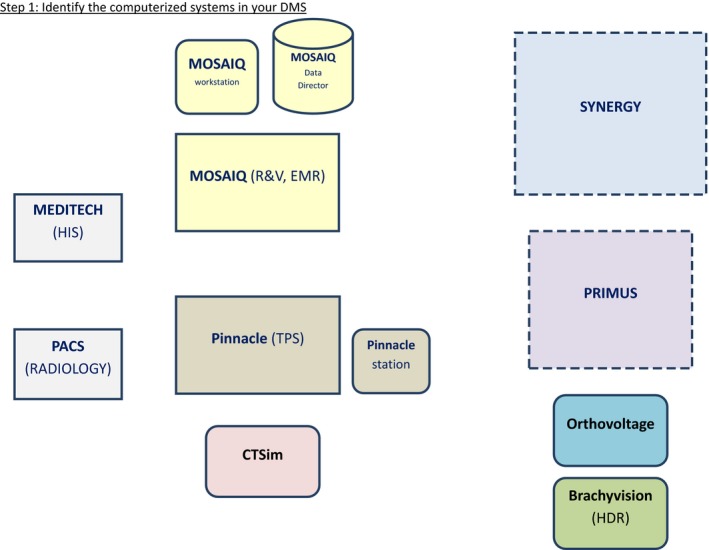
An example of components in a single site DMS (simple version)

**Figure A2:**
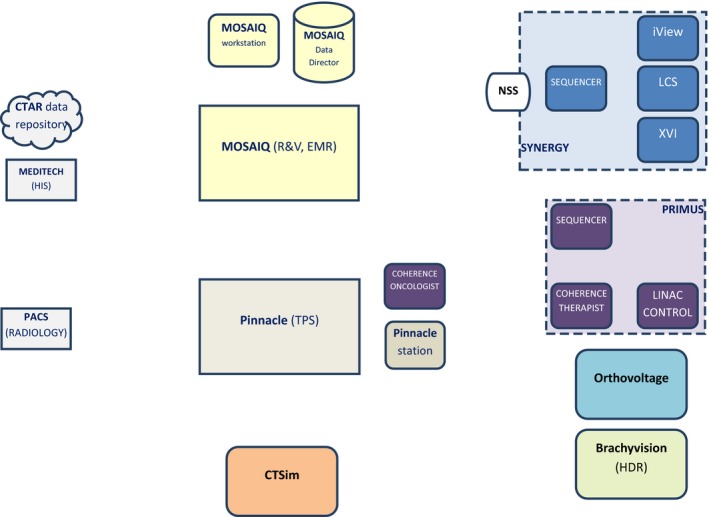
An example of components in a single site DMS (refined version)

**Figure A3:**
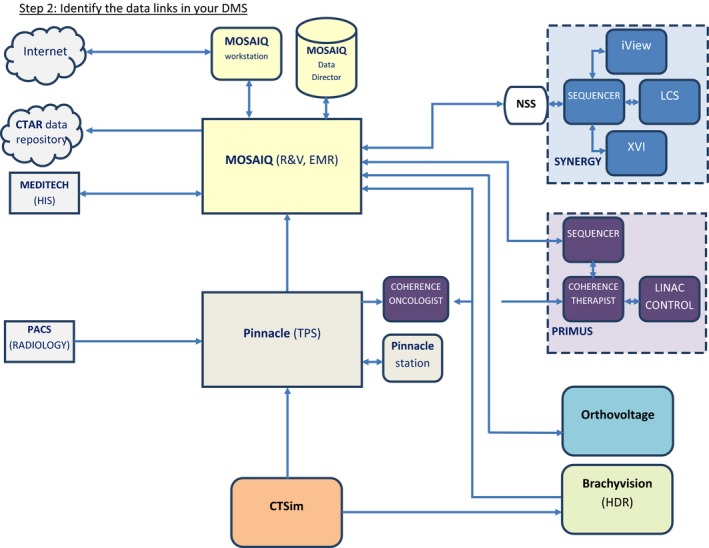
Single site DMS example: Clinical data flow.

**Figure A4:**
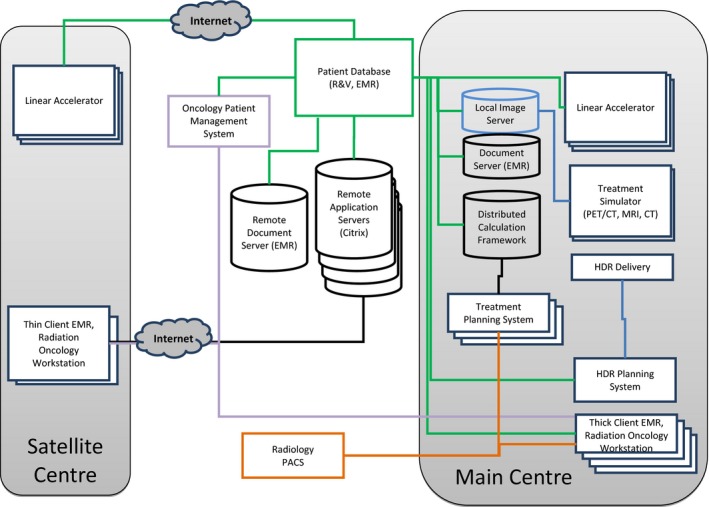
An example of a multisite DMS configuration.

**Figure A5:**
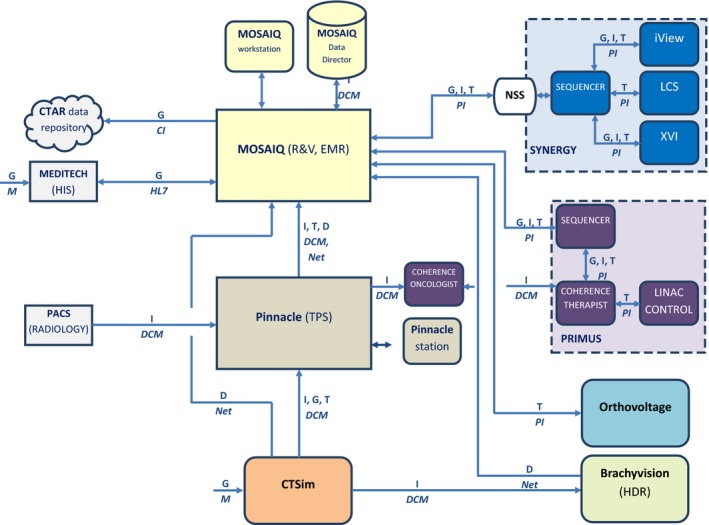
DMS System Map including data types (I: Images & imaging data, T: Treatment parameters, G: General/demographics, D: Electronic documents) and methods of data transfer (M: Manual, RM: Removable media, HL7: HL7 interface, DCM: DICOM or DICOM RT, CI: Custom interface, PI: Proprietary interface, Net: Intranet).

**Figure A6:**
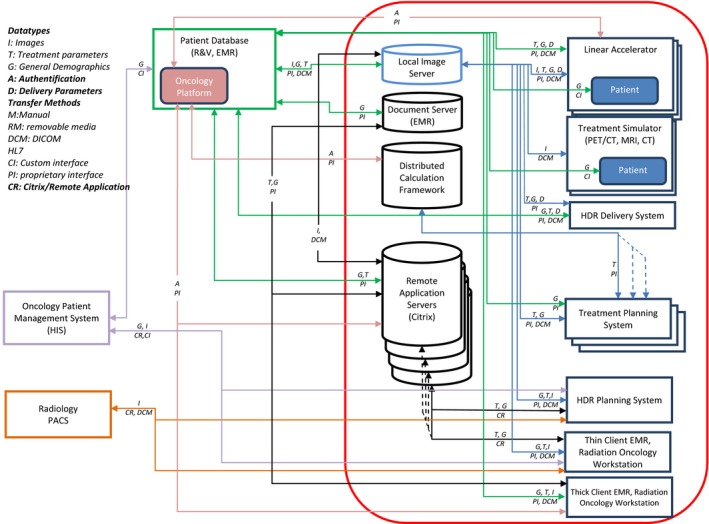
An example of a multisite DMS: System Map.

## APPENDIX 1

### METHODOLOGY FOR BUILDING A DMS QUALITY ASSURANCE PROGRAM

#### 1

This appendix provides additional information on how to develop a robust quality assurance program for a DMS.

#### STEP 1: IDENTIFY THE COMPUTERIZED SYSTEMS IN YOUR DMS

A DMS is usually composed of multiple computerized systems. The components of a DMS are specific to each center and may include one or more computerized systems from the following categories:

- Treatment delivery systems, onboard imaging systems and associated control computers, and other critical computer systems that are directly involved in delivering, monitoring, or controlling the delivery of radiation.
- Imaging systems such as CT, PET/CT, or magnetic resonance simulators and other diagnostic imaging equipment.
- Treatment planning system(s).
- Record and verify systems (R&V).
- Electronic medical record (EMR).
- Data storage servers and archival systems (e.g., MOP, MDD).
- Application servers (e.g., Citrix servers).
- Ancillary radiation oncology software within the DMS (e.g., independent monitor unit calculation software, quality assurance tools, patient safety, and event tracking systems, etc.).
- Hospital information systems (e.g., Meditech), infrastructure and network architecture.

A system map representing your DMS has a number of important functions, such as providing context in designing the testing of components within your DMS, highlighting points of potential failure and data redundancy, and helping define the roles and responsibilities of physicists, IT personnel, hospital infrastructure, and vendors within each component of the DMS. Each DMS component can be represented as a rectangle on your system map. A simple example is included in Fig. A1. When deciding on the level of detail required in your system map, consider that the goal is to represent routine clinical data flow. For example, it may be helpful to identify the individual components within a treatment delivery system and represent them separately on your system map. However, it is likely not necessary to represent individual R&V workstations on your map. Computerized systems in the department that do not handle clinical data need not be included (for example, individual computers used for email, internet access, and general desktop applications). On the other hand, remember to include systems that may be outside of your department if clinical data exchange with these systems is part of your routine process (e.g., Radiology picture archiving and communication system [PACS] or external data repositories). This can become increasingly complex with the advent of thin‐client architectures that may consist of a mixed environment of thin and thick client applications. Examples of refined system maps are included in Figs. A2 and A4.

#### STEP 2: IDENTIFY THE DATA LINKS IN YOUR DMS

Data transfer between any two computerized systems in the DMS represents a “data link.” For the purposes of quality control program design, each data link can be considered as another component in your DMS. It may be helpful to add the data links to the system map that you started in Step 1. Each link can be represented by a line in the diagram, with arrows showing the direction of data transfer (see Fig. A3 for an example).

Before choosing appropriate tests and testing frequencies, it may be helpful to categorize each data link component of your DMS based on the type or format of data exchanged as well as by the method of data transfer.

Data types or formats may include:

- **Images and imaging data (I):**
  - Medical images (DICOM images, digitally reconstructed radiographs [DRRs], CBCT, portal images, etc.)
  - Image associated data (structure sets, isocenter information, etc.)
  - Other images (e.g., setup, patient or field images in.JPG or.TIFF format)
  - Third party imaging data (respiratory/infrared/electromagnetic tracking systems)
- **Treatment delivery information (T):**
  - Radiotherapy treatment plan information (DICOM radiotherapy [DICOM RT], multileaf collimators [MLC] files, etc.)
  - Other non‐DICOM RT treatment data (beam configuration data, proprietary treatment plan information, etc.)
  - Third party data (respiratory/infrared/electromagnetic tracking systems)
  - Vendor/proprietary information
- **General/demographics (G):**
  - Health Information System (HIS) and HL7 data (demographics, etc.)
  - Laboratory, pharmacy, or other data (various formats)
- **Electronic documents (D):**
  - Electronic documents that form part of electronic medical record (e.g., PDFs, MS Word, Excel, MOSAIQ e‐SCRIBE, e‐SCAN, proprietary formats, etc.)
- **Other (O):**
  - Proprietary Pushed data (antivirus, user authentication, daemons services)
  - Proprietary Pulled data (user authentication, daemons services)

Methods/types of data exchange:

- **DICOM or DICOM RT (DCM):**
  - Import/export over network using standard DICOM or DICOM RT protocols
- **Manual entry (M):**
  - Manual entry of data into one system with reference to another system (or hard copy)
- **Interfaces (HL7, CI, PI):**
  - Standard interfaces (e.g., HL7 for demographic data transfer)
  - Custom interfaces (e.g., script for transfer of scheduling info from EMR to HIS)
  - Vendor‐specific or proprietary interfaces (e.g., image data transfer between treatment machine console and onboard imaging system)
- **Removable media (RM):**
  - Import/export from storage media (CDs, external, etc.)
- **File transfer via local Intranet (Net)**

To complete your system map, you may choose to label each data link with the data type exchanged and the method of data transfer (see Figs. A5 and A6 for examples).

#### STEP 3: CATEGORIZATION OF EACH DATA TRANSFER LINK

The most comprehensive approach to designing the quality assurance program for a DMS would include testing each of the components and data links identified in the prior two steps. In the context of limited resources, however, the responsible medical physicist may be forced to design a program of more limited scope. To ensure the highest possible effectiveness and efficiency of the quality assurance program, consider first performing a risk analysis. As suggested by the IPEM's Report 93,^3^ one possible approach is to categorize each data link by two simple criteria:

1. How important is the parameter to the treatment process?
  a Critical importance: Parameter must be transferred accurately and without delay, an error or delay directly impacts the safety of the delivered treatment.
  b Moderate importance: Parameter should be transferred, but a minor delay or error does not directly impact the safe delivery of the treatment or a work‐around is available which limits or eliminates the impact on the delivered treatment.
  c Low importance: The parameter is not necessary for the safe delivery of the treatment or a delay or error in the transfer of this parameter has no effect on the safe delivery of treatment.

Note that each center should independently assess the criticality of each parameter for the safe delivery of patient care as this is highly dependent on the configuration of a given DMS. Also note that the categorization of certain parameters may be different in an emergency vs. nonemergency treatment scenario.

1. Consider the probability or risk of failure of each data link and assign a level of “High,” “Medium,” or, “Low” risk. Factors that could lead to a higher risk of failure include: manual entry of data or wherever human error can be introduced; incomplete data transfers or exchanges (where some correction or manual entry is required); exchange based on proprietary methods that may less transparent to the user; exchange with systems outside of the clinic where many more variables may be unknown; exchange over custom interfaces (vs. “off‐the‐shelf”, rigorously tested interfaces — though these also can lead to the introduction of errors); and corrections or changes to original treatment data (requiring manual correction or re‐import of partial treatment data). The availability of support and the known stability of the data link or systems involved could also be considered. The extent of data redundancy and network rerouting capabilities in the event of catastrophic failures may also be factored in the risk analysis for more complex architectures.

A data link table can be constructed. For each data link, the sender, receiver, data type, and method of transfer can be included, as well as the assigned level of importance and level of risk. The table can then be sorted based on the combined importance and risk “score” of each data link. An example is included in Table A1.

Other risk analysis methods, such as failure mode effects analysis (FMEA) could also be utilized. Regardless of the method, the goal is to establish clear priorities for which elements of the DMS should be tested when it is not possible to develop an exhaustive program. The risk analysis also aids in establishing testing frequencies later in the quality assurance program design process, and can help define the scope of responsibilities for medical physicists, IT personnel, and vendors.

#### STEP 4: DETERMINE THE SCOPE OF THE QUALITY ASSURANCE PROGRAM

The next step of the process is to establish the scope of the DMS quality assurance program using the system map and Table 1 (DMS Data Links) in this document. Each component and data link in the DMS can now be evaluated for possible inclusion in the DMS quality assurance program.

Systems that are usually within the scope of a DMS quality assurance program:

- R&V/EMR, including application servers;
- Radiation therapy databases, storage and archival systems; and
- Any other computerized system in the radiotherapy network that handles clinical data and is excluded for the reasons outlined above.

Systems that may not be within the scope of a DMS quality assurance program:

- Treatment delivery systems and associated control computers (e.g., linear accelerators, brachytherapy afterloader and delivery systems, orthovoltage units, etc.);
- Onboard imaging systems (e.g., CBCT, portal imaging);
- Treatment simulators (CT, PET/CT, MRI);
- Other diagnostic imaging systems (Portable x ray systems, Ultrasound, etc.); and
- Treatment planning systems.

Where these systems are included in existing quality assurance programs, the physicist should evaluate whether the existing procedures cover all relevant aspects of data management and quality assurance. Where appropriate, consider additional or modified quality assurance procedures as needed (refer to Step 5). Consider that the transfer of certain data between DMS components may be validated as part of patient specific quality assurance. Where this is the case, ensure that the patient‐specific quality assurance procedure is documented and that all relevant aspects of data quality are addressed (see Step 5 for guidance on the types of tests that may apply).

Finally, identify external systems that are maintained by hospital IT staff or manufacturers through service contracts and are therefore outside the scope of your clinic's quality assurance responsibilities. Remember that application servers and hardware may be physically located elsewhere; however, if they are not independently monitored and maintained, they could still merit inclusion in your quality assurance program. Hospital information systems and network infrastructure may be beyond the scope of the clinical physicist's responsibilities. Regardless, it is important that the medical physicist be informed of the systems, configurations and procedures in place and that a multidisciplinary discussion takes place whenever hospital IT decisions, equipment servicing or third party support services could affect the clinic's DMS. A clear understanding of the scope of responsibilities for the physicists, IT personnel, hospital, and vendors is key, along with defined lines of communication when there are changes to or disruptions in service in any component of the DMS. Interdepartmental policies and procedures that formalize this communication pipeline should be in place and should be revised on an annual basis or whenever a major change to the DMS occurs.

It may be useful to update the data link and component tables to include only those elements that are within the scope of the DMS quality assurance program; however, it is recommended to document the responsible party and/or applicable quality assurance program for each element that is considered out of scope.

#### STEP 5: IDENTIFY TESTS, DETERMINE TESTING FREQUENCIES, AND ESTABLISH TOLERANCES

For each component and data link within scope, consider what tests and testing frequencies are appropriate from the list of possible test categories and examples below. The risk and importance that you associated with each data link will help you determine the extent and frequency of testing.

Note that appropriate tests and testing frequencies are highly dependent on the specific configuration and processes that govern your DMS. As such, this document cannot be prescriptive — rather it can list possible tests for data links and DMS components, and can give guidance regarding testing frequencies. It is the responsibility of the medical physicist, in collaboration with IT experts and manufacturers, to determine what is appropriate in the context of each unique DMS. An example of a resulting quality assurance program corresponding to the example DMS presented in Figs. A1, A2, A3, A4, A5, A6 and Tables A1, A2, A3, A4 is presented in Table A4.

#### Quality assurance of procedures

Quality assurance of the procedures governing the exchange of data between components of the DMS, including procedures for generating, entering, and interpreting the data. Procedures must be designed to be robust in the presence of potential data errors.^5^

End‐to‐end testing based on clinical processes is perhaps the single most important test of a DMS and should be performed at commissioning and acceptance and following a change to any component of the DMS with the potential to affect treatment data.

Rarely do physicists alone perform changes to a component of the DMS; instead, changes are initiated in partnership with vendors, local IT personnel, and often project managers. Any changes to the DMS should involve the collation of appropriate documentation prior to the work; a project plan for migrating or changing any component of the DMS, including time requirements, resource allocation, and a communication strategy; if relevant, the provision of clinical support during downtimes of the DMS; a strategy for acceptance or reintroducing affected components of the DMS into clinical service; a debriefing or compliance audit of the project; and finally modifying existing quality assurance procedures as a consequence of additional or unnecessary components and functions of the DMS.

Equally importantly, this approach requires a clear understanding of the clinical processes that rely on the DMS. Ideally, all clinical processes that generate patient data will be documented. Documentation of the clinical processes greatly facilitates standardization and documentation and standardization of processes is known to reduce errors and improve quality. Examining the documented processes in conjunction with the data management system “map” allows the development of a quality assurance program following a risk based approach.

When developing a quality assurance program for a DMS, it is important to build in mechanisms for adapting to changes — whether to a single component of the DMS, to a clinical process, or a change affecting the entire DMS. Process and system maps become obsolete quickly and it is important to maintain these as living documents.

#### Contingency planning

One of the challenges of a radiation oncology DMS is the provision for contingency planning in the event of periodic, planned, or unexpected outages. The quality assurance of the DMS from such outages is a function of the risk and frequency associated with that outage, along with the clinical needs for that centre. For example, during a scheduled DMS component upgrade when components may be offline, there may remain the need for emergent radiation therapy treatments. The inaccessibility of the patient database may limit the functionality of the R&V system such that the linear accelerator may only be used with a direct connection to the R&V system. Provisions of “offline” treatment should not only include patient treatment records, but also explore the reliance of connectivity to authentication servers and the EMR, which may or may not also be offline. Testing of such contingencies is best performed when connectivity to databases, authentication, image and document servers have planned upgrades and are expected to be offline.

The EMR may be reliant on document servers and data redundant architectures which themselves may be subjected to periodic, planned, or unexpected outages. Again, testing of back up servers and fault tolerant systems are best performed when there are planned outages. The same strategy for contingency testing holds true for inter/intranet connections between the components of the DMS.

## APPENDIX 2

### SITE‐SPECIFIC DMS QUALITY ASSURANCE PROGRAM EXAMPLE

#### 1

This appendix provides an example of how the principles of the guideline may be applied to a specific DMS.

#### STEP 3: CATEGORIZATION OF EACH DATA TRANSFER LINK

**Table A1:** Categorization of the importance and risk of data flow elements for the single site DMS example featured in Figs. A1, A2, A3, A4, A5, A6

Source	Receiver	Data Types	Method(s)	Importance	Risk of Failure
	CTSim	General	Manual	High	High
MOSAIQ	PRIMUS (SEQUENCER)	General, image, treatment parameters	Proprietary interface	High	Medium
PRIMUS (SEQUENCER)	MOSAIQ	Image, treatment parameters	Proprietary interface	High	Medium
PRIMUS (SEQUENCER)	Coherence Therapist	General, Image, Treatment Parameters	Proprietary Interface	High	Medium
Coherence Therapist	PRIMUS (SEQUENCER)	General, image, treatment parameters	Proprietary interface	High	Medium
PACS	Pinnacle	Image	DICOM	High	Medium
CTSim	Pinnacle	Image, general, treatment parameters	DICOM, DICOMRT	High	Low
Pinnacle	MOSAIQ	Image, treatment parameters, documents	DICOM, DICOMRT, intranet	High	Low
MOSAIQ	SYNERGY (SEQUENCER)	General, image, treatment parameters	Proprietary interface	High	Low
SYNERGY (SEQUENCER)	MOSAIQ	Image, treatment parameters	Proprietary interface	High	Low
SYNERGY (SEQUENCER)	SYNERGY (LCS)	Treatment parameters	Proprietary interface	High	Low
SYNERGY (LCS)	SYNERGY (SEQUENCER)	Treatment parameters	Proprietary interface	High	Low
Coherence Therapist	PRIMUS (LINAC CONTROL)	Treatment parameters	Proprietary interface	High	Low
PRIMUS (LINAC CONTROL)	Coherence Therapist	Treatment parameters	Proprietary interface	High	Low
	Meditech	General	Manual	Medium	High
MOSAIQ Data Director	MOSAIQ	Image	DICOM	Medium	Medium
Meditech	MOSAIQ	General (Demo)	HL7 interface	Medium	Low
CTSim	Brachyvision	Image	DICOM	Medium	Low
SYNERGY (SEQUENCER)	XVI	general, image	Proprietary interface	Medium	Low
XVI	SYNERGY (SEQUENCER)	Image, treatment parameters	Proprietary interface	Medium	Low
SYNERGY (SEQUENCER)	iView	General, image	Proprietary interface	Medium	Low
iView	SYNERGY (SEQUENCER)	Image, Treatment parameters	Proprietary interface	Medium	Low
Pinnacle	Coherence Oncologist	Image	DICOM	Medium	Low
Coherence Oncologist	Coherence Therapist	Image	DICOM	Medium	Low
MOSAIQ	Orthovoltage	Treatment parameters	Proprietary interface	Medium	Low
Orthovoltage	MOSAIQ	Treatment parameters	Proprietary interface	Medium	Low
MOSAIQ	CTAR	General	Custom Interface	Low	High
MOSAIQ	Meditech	General (schedule)	Custom interface	Low	Medium
MOSAIQ	MOSAIQ Data Director	Image	DICOM	Low	Medium
Brachyvision	MOSAIQ	Documents	Intranet	Low	Low

#### STEP 4: DETERMINE THE SCOPE OF THE QUALITY ASSURANCE PROGRAM

**Table A2:** Components of the DMS in Figs. A1, A2, A3, A4, A5, A6 — evaluation for inclusion in the DMS quality assurance program.

Component	Manufacturer	Notes	Relevant QA program or responsible party	Include in DMS QA program?
Meditech	Medical Information Technology, Inc.	Hospital information system (HIS)	Provincial IT	
MOSAIQ	Elekta	R&V, EMR	Performance, network and security tests are the responsibility of Provincial IT, all other aspects to be addressed by DMS quality assurance program	✓
CTAR	Accreon	Provincial data repository	Provincial IT	
Brilliance Big Bore CTSim	Philips		CTSim quality assurance program	
Pinnacle (Hardware)	Philips	Pinnacle thin client solution — servers and compute modules reside in IT department	Hardware maintenance is responsibility of Philips	
Pinnacle (TPS)	Philips	Treatment planning system	No existing quality assurance program — will be developed separately based on TQC for treatment planning systems^1^	
Brachyvision	Varian Medical Systems	Brachytherapy treatment planning system	No existing quality assurance program — will be developed separately based on TQC for treatment planning systems^1^	
GammaMed	Varian Medical Systems	Brachytherapy treatment machine	Hardware and software maintenance are the responsibility of Varian. HDR quality assurance program	
Orthovoltage	XStrahl Ltd.	Orthovoltage treatment unit	Orthovoltage quality assurance program	
SYNERGY (SEQUENCER)	Elekta	MOSAIQ station at treatment unit; communicates with linac control console via SYNERGISTIQ	Linear accelerator quality assurance program	
SYNERGY (LCS)	Elekta	Linac control console providing input to Linac Control System (LCS), operates in “Receive External Prescription” mode to receive treatment parameters from SEQUENCER	Linear accelerator quality assurance program	
SYNERGY (XVI)	Elekta	kVCBCT, communicates with SEQUENCER (including transfer of table parameters after imaging)	Linear accelerator quality assurance program	
SYNERGY (iView)	Elekta	MV Portal Imaging (EPID), auto‐forwards images to MOSAIQ, imaging analysis in MOSAIQ (SEQUENCER)	Linear accelerator quality assurance program	
PRIMUS (SEQUENCER)	Siemens	MOSAIQ station at treatment unit; communicates with linac control console via Coherence Therapist	Linear accelerator quality assurance program	
PRIMUS (Coherence Therapist)	Siemens	Communicates with linac control console, receives treatment parameters from SEQUENCER and imaging data from Coherence Oncologist	No existing quality assurance program — to be added to Linear Accelerator quality assurance program	
PRIMUS (Coherence Oncologist)	Siemens	Contouring station; receives images from Pinnacle, transfers images and contours to coherence therapist	No existing quality assurance program — to be added to Linear Accelerator quality assurance program	
PRIMUS (Linac Control)	Siemens	Receives and updates treatment parameters with coherence therapist	Linear accelerator quality assurance program	
MOSAIQ Data Director	Elekta	MOSAIQ Oncology Pacs system; archive of all DICOM image data for patients	Existing quality assurance Program	

**Table A3:** Data flow table for the DMS in Figs. A1, A2, A3, A4, A5, A6 — evaluation for inclusion in the DMS quality assurance program.

Source	Destination	Data Types	Method(s)	Importance	Risk of Failure	Relevant QA program or responsible party	Include in DMS QA program?
	CTSim	General	Manual	High	High	Manual data entry — patient‐specific quality assurance process in place	‐
MOSAIQ	PRIMUS (SEQUENCER)	General, image, treatment parameters	Proprietary interface	High	Medium	Some informal testing following upgrades or repair, and some patient‐specific quality assurance in place	**✓**
PRIMUS (SEQUENCER)	MOSAIQ	Image, treatment parameters	Proprietary interface	High	Medium	Partially covered by linear accelerator quality assurance program, image quality not addressed by existing quality assurance	**✓**
PRIMUS (SEQUENCER)	Coherence therapist	General, image, treatment parameters	Proprietary interface	High	Medium	Some informal testing following upgrades or repair, and some patient‐specific quality assurance in place, image quality not addressed by existing quality assurance	**✓**
Coherence Therapist	PRIMUS (SEQUENCER)	General, treatment Parameters	Proprietary interface	High	Medium	Some informal testing following upgrades or repair, and some patient‐specific quality assurance in place	**✓**
PACS	Pinnacle	Image	DICOM	High	Medium	Pinnacle quality assurance program	‐
CTSim	Pinnacle	Image, general, treatment parameters	DICOM, DICOMRT	High	Low	CT‐Sim quality assurance program, and patient‐specific quality assurance	‐
Pinnacle	MOSAIQ	Image, Treatment parameters, documents	DICOM, DICOMRT, intranet	High	Low	Partially addressed by Pinnacle quality assurance program as well as patient‐specific pretreatment quality assurance	**✓**
MOSAIQ	SYNERGY (SEQUENCER)	General, image, treatment parameters	Proprietary interface	High	Low	Connectivity and data transfer tested as part of linac commissioning, some limited testing following any change	**✓**
SYNERGY (SEQUENCER)	MOSAIQ	Image, treatment parameters	Proprietary interface	High	Low	Connectivity and data transfer tested as part of linac commissioning, some limited testing following any change	**✓**
SYNERGY (SEQUENCER)	SYNERGY (LCS)	Treatment parameters	Proprietary interface	High	Low	Linear accelerator quality assurance program, and connectivity and data transfer tested as part of linac commissioning	
SYNERGY (LCS)	SYNERGY (SEQUENCER)	Treatment parameters	Proprietary interface	High	Low	Linear accelerator quality assurance program, and connectivity and data transfer tested as part of linac commissioning	
Coherence Therapist	PRIMUS (LINAC CONTROL)	Treatment parameters	Proprietary interface	High	Low	Linear accelerator quality assurance program, and connectivity and data transfer tested as part of linac commissioning	
PRIMUS (LINAC CONTROL)	Coherence Therapist	Treatment parameters	Proprietary interface	High	Low	Linear accelerator quality assurance program, and connectivity and data transfer tested as part of linac commissioning	
	Meditech	General	Manual	Medium	High	Manual data entry — process quality assurance needed	
MOSAIQ Data Director	MOSAIQ	Image	DICOM	Medium	Medium	Data Director quality assurance program	
Meditech	MOSAIQ	General (Demo)	HL7 interface	Medium	Low	Connectivity and data transfer tested at interface installation, repeated following any change (responsibility of hospital IT staff), data integrity in MOSAIQ not covered by existing quality assurance	**✓**
CTSim	Brachyvision	Image	DICOM	Medium	Low	Connectivity tested at installation of Brachyvision, patient‐specific quality assurance	
SYNERGY (SEQUENCER)	XVI	General, image	Proprietary interface	Medium	Low	Linear accelerator and Onboard Imaging quality assurance programs	
XVI	SYNERGY (SEQUENCER)	Image, treatment parameters	Proprietary interface	Medium	Low	Linear accelerator and Onboard Imaging quality assurance programs	
SYNERGY (SEQUENCER)	iView	General, image	Proprietary interface	Medium	Low	Linear accelerator and Onboard Imaging quality assurance programs	
iView	SYNERGY (SEQUENCER)	Image, treatment parameters	Proprietary interface	Medium	Low	Linear accelerator and Onboard Imaging quality assurance programs	
Pinnacle	Coherence Oncologist	Image	DICOM	Medium	Low	Connectivity and data transfer tested at commissioning and following change, image quality not addressed by existing quality assurance	**✓**
Coherence Oncologist	Coherence Therapist	Image	DICOM	Medium	Low	Connectivity, image quality and data transfer tested at commissioning and following change	
MOSAIQ	Orthovoltage	Treatment parameters	Proprietary interface	Medium	Low	Connectivity and data transfer tested as part of commissioning of new control software, not fully addressed by Orthovoltage quality assurance program	**✓**
Orthovoltage	MOSAIQ	Treatment parameters	Proprietary interface	Medium	Low	Connectivity and data transfer tested as part of commissioning of new control software, not fully addressed by Orthovoltage quality assurance program	**✓**
MOSAIQ	CTAR	General	Custom interface	Low	High	Connectivity and data transfer tested at interface installation, repeated following any change (responsibility of hospital IT staff)	
MOSAIQ	Meditech	General (schedule)	Custom interface	Low	Medium	Connectivity and data transfer tested at interface installation, repeated following any change (responsibility of hospital IT staff)	
MOSAIQ	MOSAIQ Data Director	Image	DICOM	Low	Medium	Not addressed by Data Director quality assurance Program	**✓**
Brachyvision	MOSAIQ	Documents	Intranet	Low	Low	Patient‐specific check	

#### STEP 5: IDENTIFY TESTS, DETERMINE TESTING FREQUENCIES, AND ESTABLISH TOLERANCES

The specific tests required will depend highly on the infrastructure and configuration of the institution's DMS, as previously discussed. Table A4 lists some example tests for which the supervising medical physicist has decided that a weekly frequency is appropriate. The table contains samples only, and is not meant to represent a comprehensive list of tests required for the DMS. The linkage to Table 2 in this document is detailed in the associated notes.

**Table A4:** Example Test Table for the DMS in Figs. A1, A2, A3, A4, A5, A6

Designator	Test example	Performance
Weekly		
W1	Data integrity (MOSAIQ)	Complete
W2	Audit for data completeness (MOSAIQ)	Complete
W3	Monitor user and system logs	Complete

W1 **=** Patient data integrity in the MOSAIQ database is verified via the generation of custom reports focusing on known weaknesses in data transfer via interface (e.g., duplicate patients, scheduling errors, patients with alerts requiring specific measures or intervention, etc.). From designators L1 and C4 in Table 2 in this document.

W2 **=** Completeness of schedule status and workload data is verified via built‐in audit reports and/or custom reports within MOSAIQ. From designator C2 in Table 2 in this document.

W3 **=** Monitor user and system logs for unusual activity. From designator C2 in Table 2 in this document.
